# Integrating In Vitro BE Checker with In Silico Physiologically Based Biopharmaceutics Modeling to Predict the Pharmacokinetic Profiles of Oral Drug Products

**DOI:** 10.3390/pharmaceutics17091222

**Published:** 2025-09-20

**Authors:** Takuto Niino, Takato Masada, Toshihide Takagi, Makoto Kataoka, Hiroyuki Yoshida, Shinji Yamashita, Atsushi Kambayashi

**Affiliations:** 1 Faculty of Pharmaceutical Sciences, Tokyo University of Science, 6-3-1 Niijuku, Katsushika, Tokyo 125-8585, Japan; 3b25560@ed.tus.ac.jp; 2 Faculty of Pharmaceutical Sciences, Setsunan University, 45-1 Nagaotoge-Cho, Hirakata 573-0101, Osaka, Japan; takato.masada@setsunan.ac.jp (T.M.); toshihide.takagi@setsunan.ac.jp (T.T.); makoto@pharm.setsunan.ac.jp (M.K.); 3 Division of Drugs, National Institute of Health Sciences, 3-25-26 Tonomachi, Kawasaki 210-9501, Kanagawa, Japan; h.yoshida@nihs.go.jp; 4 Institute of Comprehensive Science and Engineering, Ritsumeikan University, 1-1-1 Nojihigashi, Kusatsu 525-8577, Shiga, Japan; shinji@pharm.setsunan.ac.jp; 5 Informative Bioequivalence and Bioexcellence Technology Firm, 384-5 Kuta-Kaminocho, Sakyo, Kyoto 520-0465, Kyoto, Japan

**Keywords:** BE Checker, physiologically based biopharmaceutics modeling, in vivo performance, modeling and simulation, bioequivalence

## Abstract

**Objective**: The objective of this study was to develop a Physiologically Based Biopharmaceutics Modeling (PBBM) framework that can predict PK profiles in humans based on data generated from the BE Checker. **Methods**: Metoprolol and dipyridamole were selected as model drugs. A mathematical model was developed to describe drug dissolution, membrane permeation, and dynamic changes in pH and fluid volume within the BE Checker system. Using data generated under various experimental conditions, dissolution rate constants were estimated. For dipyridamole, the precipitation rate constant was also estimated, assuming simultaneous dissolution and precipitation processes. The estimated parameters were subsequently incorporated into the human PBBM to simulate PK profiles. Finally, the predictive accuracy of PK parameters such as Cmax and AUC was assessed. **Results**: For metoprolol, the PK profiles using the paddle revolution rates of 100 and 200 rpm closely matched the observed human data, particularly for Cmax and AUC, a key indicator of BE. In the case of dipyridamole, accurate predictions of the mean human PK profile were achieved when using BE Checker data obtained under high paddle speed (200 rpm) and longer pre-FaSSIF infusion times (20–30 min). Conversely, simulations based on lower paddle speed (50 rpm) and shorter pre-FaSSIF infusion time (10 min) underestimated plasma concentrations in humans. **Conclusions**: These findings suggest that the combination of BE Checker data acquired under high agitation conditions and the in silico mathematical model developed in this study enables accurate prediction of average human PK profiles.

## 1. Introduction

Quantitative predictions of in vivo drug absorption that consider the entire absorption process have become increasingly important in drug development. This is because such predictions facilitate the evaluation of physiological factors affecting absorption and help improve development success rates through mechanistic understanding. As one such approach, we previously developed the BE Checker, an in vitro system designed to predict the absorption behavior of orally administered drug products, and demonstrated its utility in several studies [1,2]. The BE Checker simulated the gastrointestinal (GI) transit of drug products from the stomach to the small intestine by stepwise modification of the fluid’s composition, pH, and volume within a single vessel. Furthermore, by incorporating a side-by-side receiver chamber, the system enables the simultaneous assessment of membrane permeation of dissolved drug [2]. However, at present, the BE Checker can only measure the amount of dissolved drug and the extent of membrane permeation. This limitation restricts its application for bioequivalence (BE) prediction, which is the primary intended use of the system.

Physiologically Based Biopharmaceutics Modeling (PBBM), a form of Physiologically Based Pharmacokinetic (PBPK) modeling, has recently garnered increasing attention [3,4,5]. PBBM focuses on the drug absorption process in the GI tract and employs differential equations to describe various phenomena, including dosage form disintegration, drug dissolution, GI transit, and membrane permeation in the intestine [6,7,8]. PBBM enables quantitative analysis of in vivo drug behavior and allows model customization based on specific in vitro conditions [9,10]. While commercial platforms such as Simcyp and GastroPlus™ are widely used, their limited flexibility in model structure makes it difficult to fully adapt to varied in vitro test environments. Therefore, tailoring PBBMs to formulation types and in vitro experimental conditions is essential.

Previous studies have reported that the membrane permeation rate to the receiver side (% of dose) in the BE Checker is significantly lower than the in vivo fraction absorbed (Fa) [1,2]. This discrepancy may arise from differences in model assumptions—such as the use of a zero-order pre-FaSSIF (concentrated Fasted State Simulated Intestinal Fluid) infusion in the BE Checker, and the fluid volumes and hydrophilic filter specification, which do not fully reflect human physiology. Consequently, the relationship between BE Checker data and human pharmacokinetic (PK) profiles remains unclear. Moreover, systematic investigations examining the impact of in vitro BE Checker conditions—such as paddle revolution speed and FaSSIF infusion time—on predicted human PK profiles have yet to be conducted. This highlights the urgent need to establish a translational approach that links BE Checker data to PK profiles.

The objective of this study was to develop a PBBM framework that can predict average human PK profiles based on data generated from the BE Checker. Additionally, we aimed to identify the most appropriate in vitro BE Checker conditions for accurate PK prediction. To this end, we selected two model drugs: metoprolol, a BCS class 1 drug with pH-independent solubility and permeability, and dipyridamole, a weakly basic BCS class 2 drug with pH-dependent solubility. To effectively predict human PK profiles using BE Checker data, it is crucial to appropriately reproduce the dynamic fluid changes within the BE Checker. Accordingly, we developed an in silico mathematical model that incorporates drug dissolution, membrane permeation, time-dependent changes in pH, and volume fluctuations. The model was fitted to BE Checker data collected under multiple test conditions to estimate formulation-specific parameters. The estimated parameters were then incorporated into a PBBM to predict human PK profiles.

## 2. Materials and Methods

### 2.1. Chemicals and Reagents

Metoprolol tartrate (lot KYCNN-JB) and dipyridamole (lot GN01-IA) powders were purchased from Tokyo Chemical Industry Co., Ltd. (Tokyo, Japan). 3F powder^®^ (lot FFF-0723-B) was obtained from Biorelevant.com Ltd. (London, UK). Egg-phosphatidylcholine (lecithin) and sodium taurocholate (NaTC) were obtained from Fujifilm Wako Pure Chemical Corporation (Osaka, Japan) and Nacalai Tesque (Kyoto, Japan), respectively. The mixture of lecithin and NaTC was prepared through a melt-freeze cycle. Acetonitrile, isobutyl p-hydroxybenzoate, butyl p-hydroxybenzoate, sodium dihydrogen phosphate dihydrate, disodium hydrogen phosphate dodecahydrate, sodium chloride, 1 M hydrochloric acid, and 1 M sodium hydroxide solution are all analytical grade and were obtained from Kanto Chemical Co., Inc. (Tokyo, Japan).

### 2.2. Structure and Parameters of the BE Checker System

**Figure 1:**
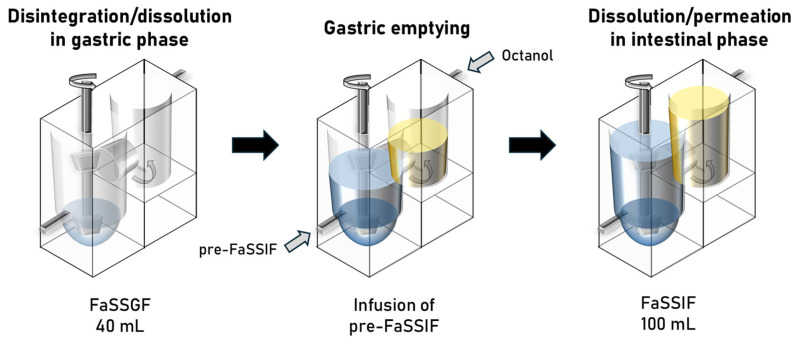
Structure and parameters of the BE Checker simulating gastrointestinal physiology in the fasted state.

The structure and parameters of the BE Checker system have been described in the previous literature [2]. Briefly, the system consists of donor and receiver chambers (Figure 1). In the donor chamber, double paddles with the diameters of 20 mm (upper) and 15 mm (lower) stir the biorelevant media: initially FaSSGF, followed by the infusion of pre-FaSSIF, and finally resulting in FaSSIF composition. The receiver chamber is filled with octanol. The fluids on both sides are infused simultaneously to maintain the same liquid level. A hydrophilic filter is mounted between the two chambers.

### 2.3. Solubility Measurement

Metoprolol is known to exhibit pH-independent dissolution and membrane permeation characteristics. Therefore, its saturated solubility was obtained from previously published literature values [11]. For dipyridamole, saturated solubility was experimentally determined using various media: FaSSGF at pH 1.6, 3.0, 5.0, and 6.5, as well as FaSSIF-V1 at pH 6.5. The preparation procedures for FaSSGF and FaSSIF-V1 were based on established protocols reported in the literature [2,12,13]. Excess amounts of dipyridamole powder were added to test tubes containing each medium. The test tubes were placed horizontally and shaken at 166 strokes per minute (stroke length: 4 cm) for 8 h at 37 °C. The sample was filtered through a 0.45 µm filter (Whatman GD/X PVDF, 13 mm, cytiva, Buckinghamshire, UK).

Dipyridamole concentrations were quantified using an isocratic HPLC system consisting of LC-20AD, LC-20AD, DGU-20A_3R_, SPD-20AV, SIL-20A, and CTO-10AS_VP_ (SHIMADZU, Kyoto, Japan). The analytical column was a TSKgel ODS-100V 5 µm (4.6 mm I.D. × 15 cm, lot 806MA00083M, Tosoh, Tokyo, Japan), maintained at 40 °C. The mobile phase comprised acetonitrile and 10 mM phosphate (Na) buffer (pH 6.9) at a ratio of 55:45 (*v*/*v*) at a flow rate of 1.0 mL/min. The injection volume was 10 µL, and the detection wavelength was 280 nm. Chromatograms were analyzed using LCsolution software (Version 1.25, SHIMADZU). The calibration curve under these analytical conditions was 0.25–75 µg/mL. All the solubility measurements were performed in triplicate.

### 2.4. Apparent Membrane Permeability Coefficient (P_app_) Measurement

In this study, the apparent membrane permeability coefficient (*P_app_*) of metoprolol and dipyridamole was experimentally evaluated [1,2]. A hydrophilic filter (Durapore^®^, 0.22 µm pore size; Merck Millipore, Burlington, MA, USA) was placed between the donor and receiver chambers (surface area: 7.6 cm^2^) of an equilibrium dialysis cell. The donor chamber (6 mL) was filled with FaSSIF-V1 containing the dissolved drug, while the receiver chamber was filled with octanol. Octanol samples were collected from the receiver side at 2, 5, 10, 15, 30, 60, 90, and 120 min after the start of the experiment. All permeability experiments were performed at 37 °C. For the quantification of metoprolol, isobutyl p-hydroxybenzoate was used as an internal standard in the HPLC analysis. The column temperature was maintained at 35 °C. The mobile phase consisted of acetonitrile and 10 mM phosphate (Na) buffer (pH 6.9) at a 65:35 (*v*/*v*) ratio, with a flow rate of 1.0 mL/min. The injection volume was 20 µL, and the detection wavelength was 224 nm. The calibration curve demonstrated linearity over the range of 0.1–200 µg/mL. For dipyridamole, the same HPLC conditions as described above were applied, except that the injection volume was 20 µL. These permeability experiments for both drugs were performed in triplicate.

The apparent membrane permeability coefficient (*P_app_*) was calculated from the linear portion of the permeation profiles: 15–60 min for metoprolol and 30–90 min for dipyridamole. The following equation was used:

(1) $$P_{app}=\frac{X_{per}}{SA\cdot C\cdot T}\;\;\;$$

where *X_per_* is the amount of drug permeated through the membrane in the linear region (μg), *SA* is the surface area of the membrane (cm^2^), *C* is the initial drug concentration in the donor chamber (μg/mL), and *T* is the sampling time interval corresponding to the linear portion (s). To evaluate the permeability of the unbound molecular form of the drug, the Henderson-Hasselbalch equation was applied under the assumption that all the drug dissolved in FaSSIF-V1 existed in free molecular form and was not incorporated into bile salt micelles.

### 2.5. In Vitro BE Checker Data

The dissolution and membrane permeability profiles of metoprolol and dipyridamole obtained in the in vitro BE Checker system were taken from a previous paper [2]. For dipyridamole, in addition to the previously reported in vitro data, new data obtained under conditions of 30 min pre-FaSSIF infusion and paddle revolution rates of 50, 100, and 200 rpm were also incorporated in the present study. For metoprolol, three experimental conditions were analyzed, each with a pre-FaSSIF infusion time of 10 min and paddle revolution rates of 50, 100, and 200 rpm. The dose of Seloken^®^ tablets (film-coated) used in the BE Checker was 20 mg. For dipyridamole, nine datasets were analyzed, combining three pre-FaSSIF infusion times (10, 20, and 30 min) with three paddle revolution rates (50, 100, and 200 rpm). These experiments were conducted under conditions of low initial pH (pH 1.6) in FaSSGF. The dose of Persantin^®^ tablets (sugar-coated) used in the BE Checker was set at 25 mg.

### 2.6. In Vivo Pharmacokinetic Data

Human PK data for metoprolol following intravenous infusion (15 mg, n = 5) and oral administration (20 mg, 50 mg, 100 mg; n = 5) were obtained from published crossover studies [14]. Similarly, human PK data for dipyridamole were obtained from crossover studies involving intravenous infusion (0.30–0.62 mg/kg) and oral administration of 100 mg (administered as four 25 mg tablets; n = 4) [15]. For both drugs, the innovator products (Seloken for metoprolol and Persantin for dipyridamole) were used in both the referenced clinical studies and the BE Checker experiments. Although minor compositional changes may have occurred over time, given the age of the clinical studies, such changes are expected to be within a range that would not influence bioequivalence. Plasma concentration-time profiles for all formulations were extracted from the published figures using WebPlotDigitizer 4.7 “https://apps.automeris.io/wpd/ (accessed on 24 December 2024)”.

### 2.7. Parameters Estimation Using the “BE Checker Model”

#### 2.7.1. “BE Checker Model” for Simulating the Dissolution and Permeation of Metoprolol from Seloken^®^ Tablets

**Figure 2:**
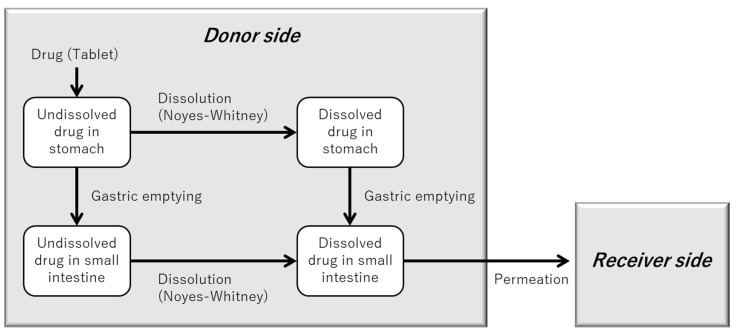
Schematic representation of the model structure describing drug dissolution and membrane permeation in the BE Checker system (for compounds without precipitation behavior).

Figure 2 illustrates the structure of the in silico model developed to represent the BE Checker environment for metoprolol.

**Figure 3:**
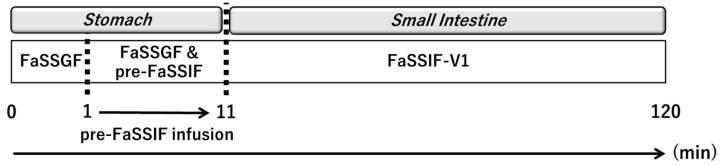
The transition from the gastric to the intestinal phase in the BE Checker model was defined as the time point at which the pre-FaSSIF infusion was completed.

Gastric emptying in the BE Checker was modeled as a zero-order infusion of pre-FaSSIF. In principle, the transition point between gastric and intestinal phases must be defined for each infusion condition within the BE Checker system. However, due to the limited number of available BE Checker datasets and the pH-independent solubility of metoprolol throughout the gastrointestinal tract [16], the impact of the gastric-to-intestinal transition timing on drug dissolution was considered negligible. Therefore, in this study, the transition from the gastric to the intestinal compartment in the model was defined as the time point at which the pre-FaSSIF infusion was completed and the FaSSIF-V1 composition was fully established (Figure 3).

The disintegration and dispersion of the formulation were not considered rate-limiting steps for drug absorption in this study. The dissolution rate of the drug in the biorelevant media and in the gastrointestinal tract under fasting conditions was assumed to follow the Noyes-Whitney equation [17,18,19,20]:

(2) $$\frac{{dW}_{t}}{dt}=z\cdot (W_{dis}+{W_{undis})}^{1/3}\cdot {W_{undis}}^{2/3}\cdot \left(Cs-\frac{W_{dis}}{V_{t}}\right)$$

where *W_dis_* is the amount of drug dissolved at time *t*, *z* is the dissolution rate constant, *W_undis_* is the amount of undissolved drug at time *t*, *Cs* is the saturation solubility in the corresponding biorelevant medium or gastrointestinal fluid, and *V_t_* is the liquid volume in the BE Checker or gastrointestinal tract at time *t*.

The membrane permeation rate of the dissolved drug into the receiver chamber of the BE Checker was modeled using the following equation:

(3) $$\frac{{dA}_{t}}{dt}=P_{app}\cdot S_{t}\cdot \frac{W_{dis}}{V_{t}}$$

where *A_t_* is the amount of drug that has permeated into the receiver side at time *t*, *S_t_* is the effective surface area of the hydrophilic filter at time *t*, and *P_app_* is the membrane permeation coefficient determined as described above. The time-dependent surface area *S_t_* of the hydrophilic filter was calculated based on the following three equations.

(4) $$h=\frac{q}{\pi \cdot R^{2}}\cdot T\;$$

(5) $$\theta =2\cdot {cos}^{-1}\cdot \left(1-\frac{h}{r}\right)$$

(6) $$St=\frac{\theta }{2}\cdot r^{2}-(r-h)\cdot \sqrt{h\cdot \left(2r-h\right)}$$

where *q* is the infusion rate of pre-FaSSIF (mL/min), *R* is the radius of the donor chamber in the BE Checker, *T* is the elapsed time after the liquid first contacts the hydrophilic filter, *h* is the height of the liquid surface in the filter region (0 cm^2^ < *h* < 6.6 cm^2^), and *r* is the radius of the hydrophilic filter.

In this study, experimental data, including saturation solubility in FaSSGF and FaSSIF, *P_app_* values, and concentration-time profiles on both the donor and receiver sides of the BE Checker were used to simultaneously fit Equations (2) and (3). The fitted parameters included z_FaSSGF_ and z_FaSSIF_, representing the dissolution rate constants under each respective condition. Model fitting was performed using Berkeley Madonna (version 10.5.1; Berkeley Madonna Inc., San Francisco, CA, USA) with the fourth-order Runge–Kutta integration algorithm.

#### 2.7.2. “BE Checker Model” for Simulating the Dissolution, Precipitation, and Permeation of Dipyridamole from Persantin^®^ Tablets

**Figure 4:**
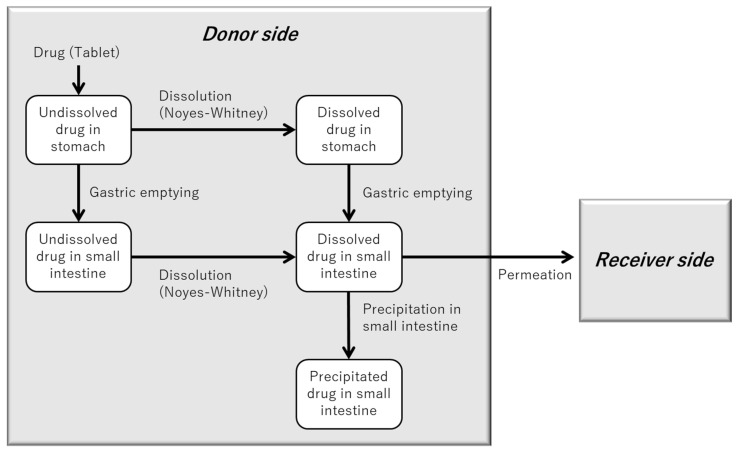
Model structure for simulating drug dissolution, precipitation, and permeation in the BE Checker system (for compounds that precipitate in the small intestine).

Figure 4 illustrates the structure of the in silico model developed to simulate the BE Checker environment for dipyridamole. Building upon the model described in Figure 2, an additional precipitation process occurring in the small intestinal phase was incorporated to account for the pH-dependent solubility and supersaturation behavior of dipyridamole.

**Figure 5:**
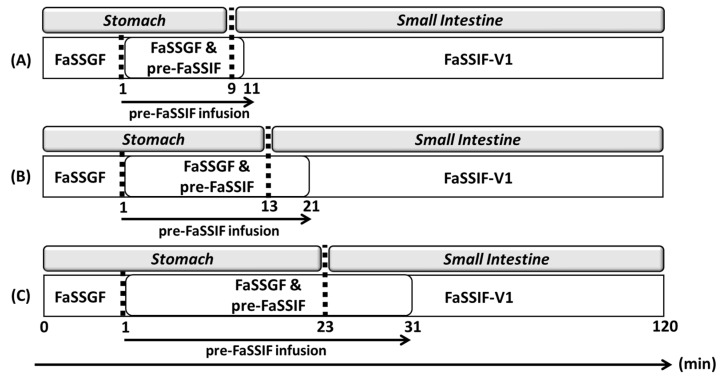
The transition from the gastric to the intestinal phase in the BE Checker model was defined as the time point at which drug precipitation was first observed in the dissolution data. (**A**) pre-FaSSIF infusion time: 10 min; (**B**) pre-FaSSIF infusion time: 20 min; (**C**) pre-FaSSIF infusion time: 30 min.

In the case of dipyridamole, the pH shift from 1.6 to 6.5 induced by pre-FaSSIF infusion resulted in drug precipitation within the BE Checker. For each pre-FaSSIF infusion condition, the onset of precipitation was defined as the transition point from the gastric to the intestinal phase in the model. The specific transition timings for each condition are summarized in Figure 5.

In the case of dipyridamole, the membrane permeation rate of the dissolved drug toward the receiver side was influenced by the fraction of drug present in its molecular (non-ionized) form, due to the proximity of its pKa (6.3) to the small intestinal pH (6.5). Additionally, the effect of bile acid concentration on membrane permeation was considered significant. Based on previous literature [21,22], the permeation process was modeled using the following equation:

(7) $$\frac{{dA}_{t}}{dt}=P_{app}\cdot f_{mol}\cdot f_{mic}\cdot SA\cdot \frac{W_{t}}{V_{t}}$$

where *f_mol_* is the fraction of drug present in the molecular form, *f_mic_* is the fraction of drug not incorporated into bile salt micelles (i.e., free drug), and *P_app_* is the intrinsic permeability coefficient derived in the above section, adjusted by dividing by (*f_mol_* × *f_mic_*). Membrane permeation in the BE Checker was thus influenced by pH and bile micelle formation during pre-FaSSIF infusion, which significantly affected the availability of free drug for permeation. A method for estimating *f_mic_* using solubility data has been proposed in the literature [23,24]. In this study, we estimated *f_mic_* based on the ratio of saturation solubilities in a neutral, micelle-free medium (FaSSGF, pH 6.5) and a micelle-containing medium (FaSSIF-V1), using the following equation:

(8) $$f_{mic}=\frac{C_{-}}{C_{+}}\;\;$$

where *C*_+_ is the saturation solubility in FaSSIF-V1 and *C*_−_ is the saturation solubility in FaSSGF (pH 6.5). It was assumed that the blank FaSSIF-V1 and the neutral FaSSGF (pH 6.5) used in the solubility experiments were comparable. Furthermore, time-dependent changes in *f_mic_* during the BE Checker experiment were modeled using a linear regression function, assuming *f_mic_* = 1.0 in FaSSGF (pH 1.6), where no micellar encapsulation occurs.

Precipitation of dipyridamole was observed in the BE Checker system. Previous studies have reported the use of “dumping” experiments to calculate concentration-dependent precipitation rate constants (*k_p_*) for weakly basic drugs and to evaluate their effects on human PK profiles [25]. In the present study, we adopted two approaches for modeling the precipitation process: one using literature-based precipitation rate constants and another using parameters estimated from actual BE Checker data. In both cases, it was assumed that once the drug had precipitated, it would not redissolve. The precipitation process was modeled using the following equation:

(9) $$\frac{{dW}_{pre}}{dt}=k_{p}\cdot (C-C_{s})\cdot V_{t}$$

where *W_pre_* is the amount of drug precipitated at time *t*, *k_p_* is the first-order precipitation rate constant, *C* is the dissolved drug concentration on the donor side at time *t*, and *C_s_* is the saturation solubility in FaSSIF-V1. Furthermore, the concentration dependence of *k_p_* was modeled using an exponential relationship derived from a prior report:

(10) $$k_{p}=k_{0}\cdot e^{X\cdot C}$$

where *k*_0_ and *X* are constants. For dipyridamole, the parameters *k*_0_ and *X* were estimated by curve fitting using Berkeley Madonna, alongside the dissolution rate constant z under each in vitro BE Checker condition.

### 2.8. PBBM for Predicting PK Profiles in Humans

#### 2.8.1. Structure of the PBBM

**Figure 6:**
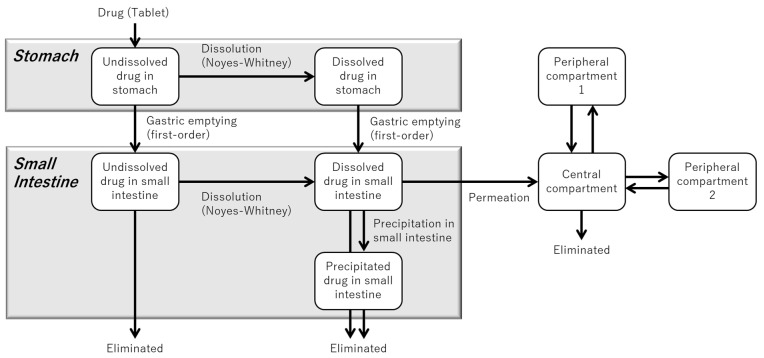
Model structure of PBBM for predicting human PK profiles.

The structure of the PBBM developed to predict human PK profiles is illustrated in Figure 6.

The PBBM was constructed and simulations were performed using Stella Professional^®^ (ver. 3.4; isee systems Inc., Lebanon, NH, USA).

The initial fluid volumes in the stomach and small intestine under fasting conditions were set to 26 mL [26,27] and 43 mL [28], respectively. Water intake was set at 240 mL (8 fl oz), based on the recommended volume in the Guidance for Industry: Bioavailability and Bioequivalence Studies by the U.S. Food and Drug Administration (FDA) [29].

Disintegration and dispersion of the formulation were assumed not to be rate-limiting for drug absorption. The dissolution rate in the gastrointestinal tract was modeled using Equation (2), and the dose-independent dissolution rate constant (*z*) [20] estimated from BE Checker data was directly applied in the PBBM without correction. It was also assumed that the in vitro z factors estimated from the BE Checker accurately reflect the in vivo dissolution rate constant *z*.

Gastric emptying of both dissolved and undissolved drugs, as well as gastric fluid, was modeled using the following first-order equation:

(11) $$\frac{{dG}_{t}}{dt}=GER\cdot X$$

where *G_t_* is the amount of drug or fluid emptied from the stomach at time *t*, *GER* is the gastric emptying rate constant, and *X* is the amount of drug or fluid remaining in the stomach. A *GER* value of 2.8 h^−1^ was used in the simulation [30]. The small intestinal transit time was set to 4 h [31]. The total gastric and small intestinal fluid volumes were constrained not to fall below their initial values during the simulation.

Precipitation in the small intestine was assumed to occur only for dipyridamole, a weakly basic compound. The precipitation process was modeled using Equation (9). Two models were developed:

Model 1 used the *k*_0_ and *X* constants estimated from BE Checker data.

Model 2 used the constant *k*_0_ (1.803 × 10^−3^ h^−1^) and *X* (38.42 mL/mg) values reported in a prior “dumping” study [25].

Drug absorption was assumed to occur only in the small intestine. The membrane permeation of dissolved drug in the small intestine into the systemic circulation was described by Equation (3). The effective surface area for absorption was set to 800 cm^2^ [32]. For metoprolol, the membrane permeation coefficient (*P_eff_*) was set to 1.34 × 10^−4^ cm/s [33]. For dipyridamole, which exhibits high permeability across intestinal epithelium [34,35], the limiting step was assumed to be diffusion across the unstirred water layer (UWL). The UWL diffusion rate constant (7.09 × 10^−4^ cm/s) was calculated using an established equation [18].

The post-absorptive PK of metoprolol were modeled using the 1-compartment model. Parameters (V_1_: 331,432 mL, K10: 0.220 h^−1^) were estimated from intravenous infusion (15 mg) PK data in humans [14] using Phoenix WinNonlin (version 8.4; Certara, L.P., Princeton, NJ, USA). The bioavailability (BA) after oral administration was calculated from the ratio of AUC_p.o._ to AUC_iv_, yielding 31%, 41%, and 46% for 20 mg, 50 mg, and 100 mg doses, respectively [14]. Given that metoprolol is rapidly and almost completely absorbed (Fa ≈ 1) [36], the intestinal and hepatic availability (Fg × Fh) was set to 0.31 (20 mg), 0.41 (50 mg), and 0.46 (100 mg).

For dipyridamole, the 2-compartment model was used to describe post-absorptive PK. Parameters (V_1_: 14,677 mL, K10: 1.16 h^−1^, K12: 1.13 h^−1^, K21: 1.34 h^−1^) were estimated from intravenous infusion data in a crossover study [15] using Phoenix WinNonlin. The volume of distribution in the central compartment was adjusted by dividing by Fg × Fh.

#### 2.8.2. Calculation of Prediction Errors for Human PK Parameters

Human PK profiles were simulated using Stella Professional^®^ by incorporating the dissolution and precipitation rate constants specific to each formulation into the PBBM framework. PK parameters, including Tmax, Cmax, and AUC, were calculated from the simulated profiles using non-compartmental analysis (NCA) in Phoenix WinNonlin. The prediction error (%*PE*) for Cmax and AUC was calculated for each formulation using the following equation:

(12) $$\%PE=\frac{\left|predicted-observed\right|}{observed}\cdot 100$$

## 3. Results and Discussion

### 3.1. Solubility

Metoprolol tartrate has been reported to exhibit consistent saturation solubility in both FaSSGF (pH 1.6) and FaSSIF-V1 (pH 6.5) [16]. In contrast, dipyridamole—whose solubility was measured in this study—demonstrated markedly higher solubility in FaSSGF (pH 1.6) compared to FaSSIF-V1 (pH 6.5), with a solubility ratio exceeding 900. This finding indicates a pronounced pH-dependent solubility profile, which is characteristic of weakly basic drugs (Table 1). Furthermore, a comparison between FaSSGF and FaSSIF-V1 at pH 6.5 revealed that the solubility of dipyridamole in FaSSIF-V1 was approximately three times greater, attributable to the presence of surfactants such as bile salts and phospholipids in the medium.

These results suggest that surfactant components, including bile acids and phospholipids, play a critical role in enhancing the dissolution of dipyridamole in the small intestinal environment. This observation is consistent with prior reports, which have emphasized the importance of dissolution media composition in the solubilization of poorly water-soluble basic compounds [37]. In particular, the dissolution behavior of BCS class 2 drugs appears to be more strongly influenced by the composition of the medium than that of BCS class 1 drugs.

### 3.2. Apparent Membrane Permeability Coefficient (P_app_)

In this study, the apparent membrane permeability coefficient (*P_app_*) of the test drugs was measured using an equilibrium dialysis cell, with octanol placed in the receiver compartment. The measured *P_app_* values were 0.268 × 10^−4^ cm/s for metoprolol and 2.27 × 10^−4^ cm/s for dipyridamole, corresponding to an approximately 8.5-fold difference. These values were calculated from the linear permeation phases of 15–60 min for metoprolol and 30–90 min for dipyridamole, using Equation (1).

Metoprolol is a basic drug with a pKa of 9.51 [38], attributed to its secondary amine functional group. Under the pH conditions used in this study (1.6–6.5), metoprolol remains predominantly in its nonionic form; however, it exhibits pH-independent solubility behavior. Moreover, no significant influence of surfactant components (e.g., bile acids or phospholipids) on its solubility has been reported [37]. Therefore, for metoprolol, correction for the free fraction (*f_mic_*) was not considered necessary in the calculation of *P_app_*.

These observed drug-to-drug ratio in *P_app_* values from this in vitro study was consistent with previously reported in vivo *P_eff_* values, which showed an approximate 5.3-fold difference between metoprolol and dipyridamole. Additionally, the ratio of in vitro *P_app_* to in vivo *P_eff_* was approximately 3- to 5-fold. This discrepancy may be attributed to differences in the effective surface area, depending on the presence or absence of microvilli.

Previous literature reported a *P_app_* value for metoprolol of 0.362 × 10^−4^ cm/s using hydrophilic membrane filters with a pore size of 0.1 μm [39], which is higher than the value obtained in this study, despite the use of a smaller pore size. This difference is likely due to variations in experimental setups (e.g., equilibrium dialysis vs. BiDP system), analytical instruments (HPLC vs. LC-MS), or testing conditions (temperature, agitation, etc.).

Caco-2 studies have reported *P_app_* values from apical to basal direction for metoprolol and dipyridamole as 0.27 × 10^−4^ and 0.16 × 10^−4^ cm/s, respectively [40,41], reversing the magnitude relationship seen in our in vitro data. These observations are likely attributable to the involvement of efflux transporters, particularly P-glycoprotein (P-gp), for which dipyridamole is a known substrate [40]. Although P-gp plays a role in limiting absorption in Caco-2 cells, its impact may be less pronounced in vivo due to saturation at drug concentrations in the gastrointestinal tract. It has also been suggested that hydrophilic filters, such as those in the BE Checker, may have limited predictive power for drugs that are transporter substrates [39]. Nonetheless, for compounds like dipyridamole, where transporter involvement is minimal at clinically relevant concentrations, the BE Checker system appears to provide a reasonable estimate of membrane permeability. For drugs whose absorption is significantly affected by transporter activity in vivo, however, more physiologically relevant systems—such as those incorporating transporter-expressing cellular membrane—may be required to improve predictive accuracy.

### 3.3. Parameter Estimation from the BE Checker Model

#### 3.3.1. Dissolution Parameter of Metoprolol in the BE Checker Model

**Figure 7:**
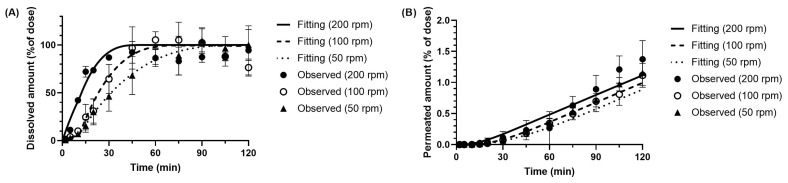
Observed and simulated profiles of (**A**) dissolved and (**B**) permeated metoprolol in the BE Checker system under the pre-FaSSIF infusion time of 10 min.

Model fitting of the in vitro data for metoprolol (Figure 7) demonstrated good agreement between the experimental data and the simulated profiles across all tested paddle speeds (50–200 rpm). The fitting results also indicated that membrane permeation to the receiver side was concentration-dependent, donor-side concentration acting as the driving force.

The model further predicted that metoprolol permeation to the receiver side would be directly proportional to the donor-side concentration. However, under the stirring conditions of 50 rpm and 100 rpm, the experimental data showed an unexpected reversal in the relationship between donor-side dissolution and receiver-side permeation. This phenomenon is likely attributable to the membrane permeability characteristics of metoprolol, which limit the experimental detection of differences in receiver-side profiles, even when dissolution rates vary. Consequently, variations in dissolution profiles are not readily reflected in the membrane permeation profiles.

Table 2 represents the dissolution rate constant z estimated from the BE Checker data. The results show that small intestinal z-factor increased with increasing paddle speed, whereas the gastric z-factors were comparable between 50 and 100 rpm. Additionally, in all tested conditions, the z-factors in the small intestine were consistently higher than those in the stomach.

#### 3.3.2. Dissolution and Precipitation Parameters of Dipyridamole in the BE Checker Model

**Figure 8:**
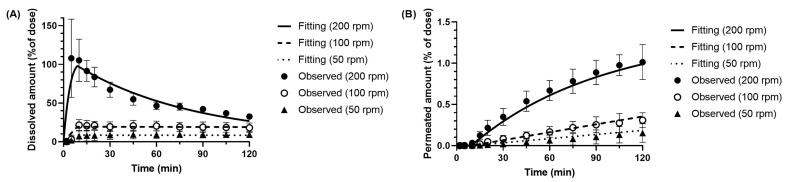
Observed and simulated profiles of (**A**) dissolved and (**B**) permeated dipyridamole in the BE Checker system under the pre-FaSSIF infusion time of 10 min.

**Figure 9:**
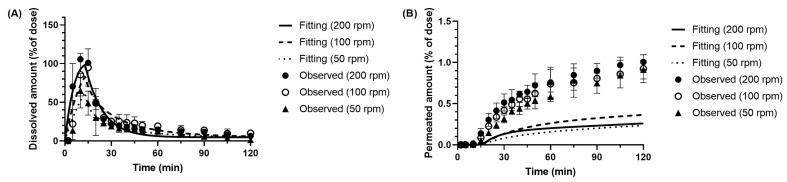
Observed and simulated profiles of (**A**) dissolved and (**B**) permeated dipyridamole in the BE Checker system under the pre-FaSSIF infusion time of 20 min.

**Figure 10:**
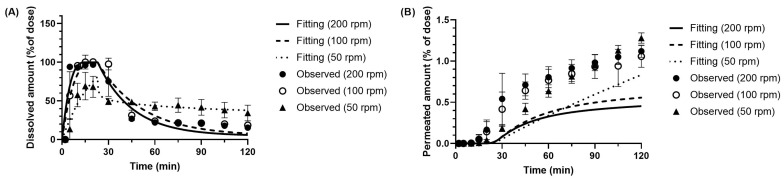
Observed and simulated profiles of (**A**) dissolved and (**B**) permeated dipyridamole in the BE Checker system under the pre-FaSSIF infusion time of 30 min.

The fitting results of the BE Checker model for dipyridamole demonstrated good agreement between the simulated and observed donor-side dissolution profiles (including precipitation) under all tested conditions. In particular, under the 10 min pre-FaSSIF infusion condition (Figure 8), both dissolution and membrane permeation profiles were well reproduced by the model. In contrast, under the 20 and 30 min infusion conditions (Figure 9 and Figure 10), the simulated membrane permeation profiles were lower than the experimentally measured values.

Since the donor-side concentrations were substantially higher than those on the receiver side in these experiments, the permeation kinetics estimated through model fitting were primarily driven by the dissolution profile on the donor side. Consequently, in the 10 min infusion condition—where stirring speed had a pronounced effect on dissolution behavior—the model could accurately reflect the relationship between donor concentration and receiver-side permeation, resulting in a good fit. However, under the 20 and 30 min infusion conditions, two factors likely contributed to the reduced fitting accuracy: (i) The BE Checker system’s limitations in capturing the initial dissolution behavior, as previously noted in the metoprolol analysis; and (ii) The inability to experimentally confirm fine differences in membrane permeability that depend on temporal fluctuations in dissolved drug concentration under prolonged infusion.

The BE Checker model developed in this study incorporated the effect of bile acid concentration on membrane permeability using the solubility-based method. Specifically, *P_app_* was estimated from the equilibrium concentration in a FaSSIF-V1-filled system. However, during the pre-FaSSIF infusion process, the actual *P_app_* may vary over time due to changes in concentration gradients and differences in solvent composition, which were not fully captured in the model. This deviation is likely more pronounced under prolonged infusion conditions (20–30 min), and may have contributed to the discrepancies between predicted and observed permeation profiles. Despite these limitations, the dissolution data were well fitted under all conditions, even though apparent precipitation occurred under many of them. Therefore, the dissolution rate and precipitation rate parameters derived from this model are considered to be reliable for further application in PBBM.

The results presented in Table 3 indicate a general trend of increasing gastric z-factors with higher paddle revolution speeds across all pre-FaSSIF infusion time conditions. In contrast, no clear correlation was observed between small intestinal z-factors or precipitation rate constants. Notably, under the 30 min pre-FaSSIF infusion condition at 100 rpm, the estimated small intestinal z-factor was anomalously high compared to the other conditions.

In this study, four parameters were simultaneously estimated from BE Checker dissolution and membrane permeation data: the gastric and intestinal dissolution rate constants (z) and the precipitation rate constants (k_0_, X). However, the multiparameter estimation approach is subject to certain limitations. Specifically, simultaneous fitting of multiple independent parameters can result in overfitting, thereby reducing the reliability of individual estimates—particularly for the small intestine z factor and precipitation rate constants. The unusually high small intestinal z-factor observed under the 30 min infusion, 100 rpm condition is likely due to this limitation.

A previous study [42] reported that dipyridamole dissolves rapidly and completely under acidic conditions (FaSSGF, pH 1.6), achieving 100% dissolution within 10 min. In contrast, under the FaSSIF-V1 condition (pH 6.5), the dissolution rate is substantially lower, with only ~15% of the drug dissolved after 120 min. These findings suggest that gastric dissolution plays a major role in determining the systemic exposure of dipyridamole, whereas small intestinal dissolution and precipitation have a relatively minor impact on its PK profile [42,43]. Accordingly, for weakly basic drugs such as dipyridamole—where gastric dissolution is the primary determinant of oral absorption—errors in the estimation of small intestinal z or precipitation rate constants are unlikely to significantly affect PK predictions. However, for basic drugs in which small intestinal dissolution and precipitation play a substantial role in absorption, such estimation inaccuracies may not be negligible. To address this issue, two strategies may be considered. First, integrating BE Checker data across multiple dosing levels could improve the robustness of parameter estimation by introducing constraints on parameter ranges. Second, increasing the membrane permeation clearance—e.g., by enlarging the surface area-to-volume (SA/V) ratio of the in vitro system—could enhance model sensitivity to concentration gradients. However, replacing the human SA/V ratio in vitro is technically challenging and may not be feasible with current experimental platforms.

### 3.4. Predicted Pharmacokinetic Profiles in Humans

#### 3.4.1. Predicted PK Profiles of Metoprolol

**Figure 11:**
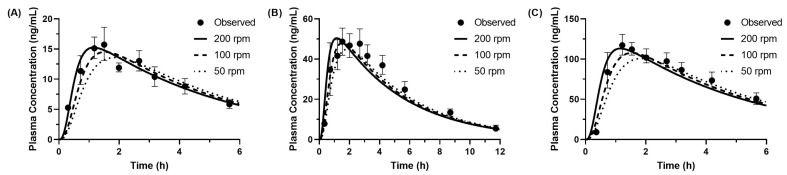
Observed and predicted pharmacokinetic profiles of metoprolol following oral administration of (**A**) 20 mg, (**B**) 50 mg, and (**C**) 100 mg Seloken^®^ Tablets. Each profile was predicted using in vitro BE Checker data with the pre-FaSSIF infusion time of 10 min and the paddle revolution rates ranging from 50 to 200 rpm.

Using the dissolution rate constants z for the stomach and small intestine shown in Table 2, human PK profiles were predicted using PBBM for 20 mg, 50 mg, and 100 mg oral doses of Seloken^®^ Tablets. The predicted PK profiles are shown in Figure 11, and the corresponding PK parameters are summarized in Table 4.

Analysis of each condition revealed that under the revolution speed of 50 rpm, the prediction error for Cmax ranged from −15% to −9%, which was slightly less accurate than the 100 rpm and 200 rpm (−9% to +2%). A consistent trend of improved Cmax prediction accuracy was observed with increasing paddle speed. Although the prediction error at 20 mg was slightly smaller, no substantial dose-dependent differences were noted. The prediction error for AUC_inf_ at paddle speeds of 50, 100, and 200 rpm was +7%, −11%, and −6%, respectively, demonstrating relatively comparable accuracy across stirring conditions. The Tmax prediction error ranged from +0.28 h to +0.66 h at 50 rpm, +0.05 to +0.32 h at 100 rpm, and −0.44 to −0.06 h at 200 rpm. These results indicate a tendency for Tmax to decrease with increasing paddle speed, consistent across all doses.

Overall, the analysis confirmed that paddle speeds of 100 and 200 rpm yielded accurate predictors for Cmax and AUC_inf_, key parameters used in bioequivalence (BE) evaluation. Notably, Cmax prediction accuracy clearly improved with increasing agitation speed. This suggests that gastric dissolution (0–11 min) is sufficiently accelerated under stirring conditions of 100 rpm or higher to closely mimic in vivo drug release. In contrast, the underprediction of Cmax at 50 rpm, despite reasonably accurate AUC_inf_, may be attributed to delayed dissolution in the BE Checker relative to the in vivo dissolution rate. This likely resulted in slower initial absorption, affecting Cmax. These findings emphasize the critical role of paddle revolution speed in determining dissolution kinetics of metoprolol, particularly during the initial dissolution profile.

Based on these results, a paddle revolution speed of at least 100 rpm is recommended to best replicate the average human gastrointestinal environment. In particular, accurately reproducing the initial dissolution phase is crucial for reliable Cmax prediction. The findings may serve as a generalizable principle for BE evaluation of other BCS Class 1 drugs, and provide valuable guidance for optimizing in vitro evaluation systems in the future formulation development.

#### 3.4.2. Predicted PK Profiles of Dipyridamole (Model 1)

**Figure 12:**
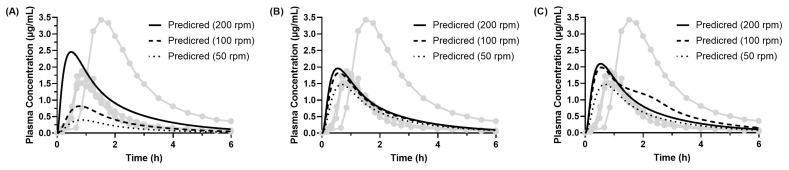
Observed (individual, n = 4; gray lines in the figure) and predicted PK profiles of dipyridamole following oral administration of 100 mg Persantin^®^ Tablets. Each plasma concentration-time profile was simulated using in vitro BE Checker data corresponding to pre-FaSSIF infusion times of (**A**)10 min, (**B**) 20 min, and (**C**) 30 min.

For dipyridamole, human PK data (n = 4) following 100 mg oral administration (four 25 mg tablets) from a crossover study were used [15]. The model input parameters were estimated by curve-fitting the in vitro BE Checker data, and applied to the PBBM to simulate the human PK profile (Model 1). The predicted PK profile is shown in Figure 12, and the predicted parameters are summarized in Table 5.

Assuming a prediction error (PE) within ±15% to represent good predictive accuracy, the model accurately predicted both Cmax (−10% to −4%) and AUC_inf_ (−5% to +1%) under the conditions of pre-FaSSIF infusion times of 20 or 30 min combined with a paddle revolution speed of 200 rpm. Under other conditions, the PE exceeded ±15% for either Cmax or AUC_inf_. In particular, the poorest prediction accuracy was observed with a pre-FaSSIF-infusion time of 10 min and paddle speed of 50 rpm, with PE values of −82% for Cmax and −77% for AUC_inf_. The low paddle speed of 50 rpm yielded poor predictions for both Cmax (−82% to −30%) and AUC_inf_ (−77% to −24%), regardless of pre-FaSSIF infusion time.

These results indicate that a pre-FaSSIF infusion time of 20–30 min and a paddle speed of 200 rpm are optimal for accurately predicting the human PK profiles of dipyridamole. This condition likely better mimics the physiological environment of the human gastrointestinal tract. In particular, the relatively vigorous agitation at 200 rpm may better simulate gastric motility, enabling more accurate assessment of dissolution kinetics.

In contrast, the diminished prediction accuracy under the 10 min infusion condition may be attributed to dipyridamole’s strong pH-dependent solubility. The rapid transition from acidic (gastric) to neutral (intestinal) pH may not have been adequately reproduced in vitro. The shortened exposure to acidic media likely led to underestimation of the gastric dissolution rate constant z, resulting in deviation from in vivo conditions. Furthermore, the limited data points around the precipitation onset (approx. 9 min) may have hindered accurate curve-fitting.

Under the condition of 30 min infusion and a paddle speed of 100 rpm, the model notably overpredicted AUC_inf_ (+21.5%), possibly due to overestimation of small intestinal z-factor, resulting in prolonged absorption (~3 h). Although predictions of Cmax and AUC_inf_ were accurate under optimal conditions, Tmax was consistently underestimated by approximately 0.50 h. This may reflect variability among subjects, with some showing Tmax values around 1.5 h, or the model’s inability to reproduce lag time for all individuals.

Taken together, these findings suggest that artificial gastric fluid (pH 1.6), paddle speed of 200 rpm, and pre-FaSSIF infusion time of 20–30 min are optimal for BE Checker-based prediction of dipyridamole’s human PK profile. Further studies should validate these optimal conditions with additional experiments and improve the model by incorporating a multi-dose data fitting strategy. These insights may inform biopharmaceutics evaluation for other BCS Class 2b drugs with similar dissolution characteristics.

#### 3.4.3. Predicted PK Profiles of Dipyridamole (Model 2)

**Figure 13:**
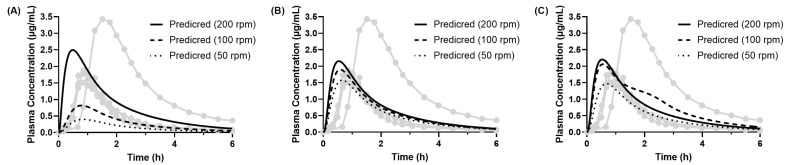
Observed (individual, n = 4; gray lines in the figure) and predicted PK profiles of dipyridamole following oral administration of 100 mg Persantin^®^ Tablets. Predictions were generated using the PBBM (Model 2). Each plasma concentration was predicted using in vitro BE Checker data with the pre-FaSSIF infusion time of (**A**) 10 min, (**B**) 20 min, and (**C**) 30 min.

For dipyridamole, Model 2 was constructed by applying the precipitation rate constants obtained from a separate “dumping” experiment reported in the literature [25]. In this model, the dissolution rate parameters were assumed to be the same as those obtained in Model 1, as we focused on the effect of different precipitation values on the predicted PK profiles. The predicted human PK profiles are shown in Figure 13, and the corresponding PK parameters are summarized in Table 6.

The results indicate that the BE Checker conditions of a pre-FaSSIF infusion time of 20 min (at 100 and 200 rpm) and 30 min (at 200 rpm) provided accurate predictions of human PK profiles in terms of both Cmax and AUC_inf_. Under other conditions, the prediction results were generally consistent with those obtained using Model 1. Overall, no substantial difference was observed in prediction accuracy between Model 2 (which uses precipitation parameters derived from the dumping study) and Model 1 (which uses those estimated from BE Checker data). However, Model 2 yielded slightly improved prediction accuracy under some conditions, particularly with a 20 min pre-FaSSIF infusion time and paddle speeds of 50–200 rpm. Across all BE Checker conditions, the predicted Tmax tended to be shorter than the observed values, And the magnitude of Tmax prediction errors was similar between the two models.

According to previous reports [43], minimal precipitation (<10%) of dipyridamole occurs in the upper small intestine in humans, suggesting that in vivo precipitation is negligible. Another study [25] has shown that rapid gastric emptying and high intragastric drug concentrations may increase the likelihood of precipitation in the small intestine. These findings suggest that Model 1 is suitable for predicting average human PK profiles in cases where in vivo precipitation has a minimal impact. In contrast, for drugs or conditions where precipitation significantly affects absorption, the precipitation rate constants used in Model 1 may overestimate the extent of in vivo precipitation. In such cases, Model 2, which incorporates precipitation kinetics derived from dumping experiments, may provide more reliable predictions.

In both models, a general trend toward earlier Tmax predictions was observed. This discrepancy may be attributed to interindividual variability in Tmax (ranging from 0.83 to 1.51 h) as well as subject-specific lag times noted in the reported human data [15].

### 3.5. Discussion Based on the Overall Results

In this study, a comprehensive in vitro-in silico evaluation protocol was developed using the BE Checker system, with metoprolol and dipyridamole serving as model drugs. The primary finding was that the optimized test conditions—simulated gastric fluid at pH 1.6, a paddle revolution speed of 200 rpm, and pre-FaSSIF infusion time of 20 to 30 min—enabled accurate prediction of the average human PK profiles, particularly Cmax and AUC_inf_, for both compounds. When these conditions were incorporated into the PBBM constructed in this study, the in vivo performance of formulations could be predicted with high accuracy. This integrated approach offers a promising framework for enhancing the understanding of the correlation between in vitro dissolution/permeation kinetics and in vivo PK profiles.

Looking forward, this system provides a strong foundation for implementing virtual bioequivalence (VBE) studies, as it allows for parameter customization that accounts for inter-individual variability (e.g., intragastric pH, bile acid concentration, buffer capacity). In the in vitro BE Checker experiment, the characteristics of FaSSGF and FaSSIF can be adjusted to reflect inter-individual variability, and modifying the pre-FaSSIF infusion period can represent variability in the gastric emptying rate. In the in silico model, parameters such as fluid volume and gastric emptying rate can also be adjusted to reflect individual physiology. Such capabilities are expected to contribute to more precise BE predictions during formulation development and to enable personalized formulation strategies for special populations such as pediatric, geriatric, or diseased individuals with altered gastrointestinal physiology. The protocol established here may support the advancement of formulation science by improving the efficiency of the development workflow and offering a new standard for in vitro-in vivo correlation (IVIVC).

It is important to note that the hydrodynamic conditions used in the BE Checker differ fundamentally from those of conventional (compendial) dissolution systems such as USP Apparatus 2 (paddle method). Specifically, the BE Checker vessel has a small diameter (4.6 cm) and volume (100 mL) compared to those used in USP methods. As a result, the hydrodynamic forces generated at 200 rpm in the BE Checker are not directly comparable to those of the same paddle speed in USP Apparatus 2. Thus, 200 rpm in the BE Checker does not correspond to 200 rpm in USP Apparatus 2, and caution should be exercised when interpreting or comparing these conditions.

Several challenges remain for the broad application of this system across different drugs and patient populations. One major limitation is the accuracy of parameter fitting. To improve model robustness, future work should incorporate simultaneous analysis of multi-dose datasets. This approach is expected to enhance the precision of parameter estimation and increase the reliability of PK predictions.

In the current study, human PK profiles were predicted for healthy average adults. However, extension to VBE and special population modeling will require a customized BE Checker and in silico conditions that more accurately reflect the physiological variability among individuals.

Additionally, the accurate determination of the effective permeability (*P_eff_*) remains a key issue. In this study, human *P_eff_* values for metoprolol and dipyridamole were sourced from the literature. However, when applying this system to the development of new chemical entities, such data are typically unavailable. Scaling from *P_app_* values derived from equilibrium dialysis or hydrophilic filter systems to in vivo *P_eff_* remains challenging. This challenge is particularly evident for low-permeability drugs (BCS classes 3 and 4), compared with the high-permeability drugs used in the present study. Therefore, further research should focus on the development of reliable methods for predicting human *P_eff_*.

## 4. Conclusions

In this study, a physiologically based biopharmaceutics model (PBBM) was developed to predict the average human PK profile of drugs based on dissolution and membrane permeation data obtained using the BE Checker. This approach successfully enabled the construction of a PBBM capable of predicting in vivo PK profiles and led to the identification of optimal reference conditions for in vitro testing with the BE Checker. Moreover, the modeling strategy employed in the present study may serve as a valuable framework, and the overall in silico concept could be extended to other in vitro systems for predicting the in vivo performance of orally administered dosage forms.

Furthermore, several key considerations for improving prediction accuracy were identified, including parameter fitting methodology, accounting for inter-individual variability in gastrointestinal physiology, and the accurate estimation of effective permeability (*P_eff_*).

The findings of this study provide foundational knowledge that supports the future implementation of “virtual bioequivalence studies” and the prediction of drug absorption in special populations. By incorporating individual physiological variability—such as intragastric pH, bile acid concentration, and buffer capacity—this approach offers a promising path toward more precise and personalized drug development.

## Figures and Tables

**Table 1:** Saturation solubility of metoprolol tartrate and dipyridamole in various biorelevant media.

Drug	Biorelevant Media	Solubility (Mean ± SD)
Metoprolol tartrate	SGF and FaSSIF (pH 1.6~pH 6.5)	>2 mg/mL [11,16]
Dipyridamole	FaSSGF (pH 1.6)	11.16 ± 0.15 mg/mL
	FaSSGF (pH 3.0)	0.304 ± 0.063 mg/mL
	FaSSGF (pH 5.0)	0.0072 ± 0.0015 mg/mL
	FaSSGF (pH 6.5)	0.0043 ± 0.0005 mg/mL
	FaSSIF-V1 (pH 6.5)	0.0118 ± 0.0002 mg/mL

**Table 2:** Estimated dissolution rate constant (z) for metoprolol under various BE Checker conditions.

BE Checker Condition (Pre-FaSSIF Infusion Time, Stirring Speeds)	*z* Factor (mL·mg^−1^·min^−1^)	
10 min, 50 rpm	Stomach	3.74 × 10^−4^
	Small intestine	1.60 × 10^−3^
10 min, 100 rpm	Stomach	3.54 × 10^−4^
	Small intestine	2.69 × 10^−3^
10 min, 200 rpm	Stomach	2.89 × 10^−3^
	Small intestine	3.79 × 10^−3^

**Table 3:** Estimated dissolution and precipitation parameters of dipyridamole under various BE Checker conditions.

BE Checker Condition (Pre-FaSSIF Infusion Time, Stirring Speeds)	*z* Factor (mL·mg^−1^·min^−1^)		*k*_0_ (min^−1^)	*X* (mL/mg)
10 min, 50 rpm	Stomach	8.77 × 10^−4^	8.67 × 10^−19^	4.24
	Small intestine	3.94 × 10^−13^		
10 min, 100 rpm	Stomach	2.07 × 10^−3^	1.34 × 10^−18^	1.83 × 10^−13^
	Small intestine	9.17 × 10^−15^		
10 min, 200 rpm	Stomach	6.52 × 10^−2^	1.28 × 10^−2^	6.64 × 10^−13^
	Small intestine	6.17 × 10^−16^		
20 min, 50 rpm	Stomach	5.85 × 10^−3^	2.64 × 10^−2^	10.8
	Small intestine	1.16 × 10^−11^		
20 min, 100 rpm	Stomach	9.44 × 10^−3^	3.03 × 10^−2^	4.42
	Small intestine	1.00 × 10^−14^		
20 min, 200 rpm	Stomach	1.58 × 10^−2^	8.75 × 10^−2^	1.85 × 10^−21^
	Small intestine	6.32 × 10^−14^		
30 min, 50 rpm	Stomach	5.09 × 10^−3^	1.85 × 10^−5^	48.5
	Small intestine	1.61 × 10^−9^		
30 min, 100 rpm	Stomach	1.27 × 10^−2^	3.46 × 10^−2^	2.63 × 10^−13^
	Small intestine	8.82		
30 min, 200 rpm	Stomach	1.79 × 10^−2^	4.43 × 10^−2^	6.60 × 10^−14^
	Small intestine	3.38 × 10^−15^		

**Table 4:** Observed and predicted pharmacokinetic parameters of metoprolol following oral administration of 20 mg, 50 mg, and 100 mg Seloken^®^ Tablets.

Dose (mg)		Observed	Predicted (50 rpm)	Predicted (100 rpm)	Predicted (200 rpm)
20	Tmax (h)	1.53	1.86 (+0.33 h)	1.53 (+0.00 h)	1.15 (−0.38 h)
	Cmax (ng/mL)	16.0	13.6 (−15%)	14.5 (−9%)	15.3 (−5%)
	AUC_inf_ (ng·h/mL)	79.9	85.7 (+7%)	85.4 (+7%)	85.4 (+7%)
50	Tmax (h)	1.59	1.86 (+0.28 h)	1.54 (−0.05 h)	1.15 (−0.44 h)
	Cmax (ng/mL)	49.2	44.9 (−9%)	48.0 (−3%)	50.4 (+2%)
	AUC_inf_ (ng·h/mL)	318	282 (−11%)	282 (−11%)	282 (−11%)
100	Tmax (h)	1.22	1.87 (+0.66 h)	1.54 (+0.32 h)	1.15 (−0.06 h)
	Cmax (ng/mL)	117	101 (−14%)	108 (−8%)	113 (−3%)
	AUC_inf_ (ng·h/mL)	677	636 (−6%)	634 (−6%)	633 (−6%)

**Table 5:** Observed and predicted PK parameters of dipyridamole following oral administration of 100 mg Persantin^®^ Tablets.

	Observed (Mean ± SD)	Predicted								
		10 min			20 min			30 min		
		50 rpm	100 rpm	200 rpm	50 rpm	100 rpm	200 rpm	50 rpm	100 rpm	200 rpm
Tmax (h)	1.03 ± 0.33	0.86 (−0.18 h)	0.79 (−0.24 h)	0.49 (−0.55 h)	0.66 (−0.37 h)	0.59 (−0.44 h)	0.55 (−0.48 h)	0.68 (−0.36 h)	0.56 (−0.47 h)	0.54 (−0.50 h)
Cmax (μg/mL)	2.18 ± 0.85	0.40 (−82%)	0.82 (−62%)	2.46 (+13%)	1.52 (−30%)	1.82 (−16%)	1.96 (−10%)	1.47 (−32%)	1.98 (−9%)	2.09 (−4%)
AUC_inf_ (μg·h/mL)	4.25 ± 2.73	0.96 (−77%)	1.89 (−56%)	4.98 (+17%)	3.23 (−24%)	3.78 (−11%)	4.05 (−5%)	3.14 (−26%)	5.16 (+22%)	4.29 (+1%)

**Table 6:** Observed and predicted PK parameters of dipyridamole following oral administration of 100 mg Persantin^®^ Tablets (Model 2).

	Observed (Mean ± SD)	Predicted								
		10 min			20 min			30 min		
		50 rpm	100 rpm	200 rpm	50 rpm	100 rpm	200 rpm	50 rpm	100 rpm	200 rpm
Tmax (h)	1.03 ± 0.33	0.86 (−0.18 h)	0.79 (−0.24 h)	0.49 (−0.55 h)	0.66 (−0.38 h)	0.59 (−0.45 h)	0.54 (−0.49 h)	0.68 (−0.36 h)	0.56 (−0.48 h)	0.53 (−0.50 h)
Cmax (μg/mL)	2.18 ± 0.85	0.40 (−82%)	0.82 (−62%)	2.50 (+15%)	1.58 (−28%)	1.90 (−13%)	2.15 (−1%)	1.47 (−32%)	2.06 (−5%)	2.20 (+1%)
AUC_inf_ (μg·h/mL)	4.25 ± 2.73	0.96 (−77%)	1.89 (−56%)	5.04 (+19%)	3.32 (−22%)	3.90 (−8%)	4.38 (+3%)	3.14 (−26%)	5.29 (+25%)	4.47 (+5%)

## Data Availability

The original contributions presented in this study are included in the article. Further inquiries can be directed to the corresponding author.

## References

[1] Takagi T., Masada T., Minami K., Kataoka M., Yamashita S. (2023). Development of an In Vitro Methodology to Assess the Bioequivalence of Orally Disintegrating Tablets Taken without Water. Pharmaceutics.

[2] Masada T., Takagi T., Minami K., Kataoka M., Izutsu K., Matsui K., Yamashita S. (2021). Bioequivalence of Oral Drug Products in the Healthy and Special Populations: Assessment and Prediction Using a Newly Developed In Vitro System “BE Checker”. Pharmaceutics.

[3] Riedmaier A.E., DeMent K., Huckle J., Bransford P., Stillhart C., Lloyd R., Alluri R., Basu S., Chen Y., Dhamankar V. (2020). Use of Physiologically Based Pharmacokinetic (PBPK) Modeling for Predicting Drug-Food Interactions: An Industry Perspective. AAPS J..

[4] Heimbach T., Kesisoglou F., Novakovic J., Tistaert C., Mueller-Zsigmondy M., Kollipara S., Ahmed T., Mitra A., Suarez-Sharp S. (2021). Establishing the Bioequivalence Safe Space for Immediate-Release Oral Dosage Forms Using Physiologically Based Biopharmaceutics Modeling (PBBM): Case Studies. J. Pharm. Sci..

[5] Kollipara S., Martins F.S., Sanghavi M., Santos G.M.L., Saini A., Ahmed T. (2024). Role of Physiologically Based Biopharmaceutics Modeling (PBBM) in Fed Bioequivalence Study Waivers: Regulatory Outlook, Case Studies and Future Perspectives. J. Pharm. Sci..

[6] Kato T., Mikkaichi T., Yoshigae Y., Okudaira N., Shimizu T., Izumi T., Ando S., Matsumoto Y. (2021). Quantitative Analysis of an Impact of P-Glycoprotein on Edoxaban’s Disposition Using a Human Physiologically Based Pharmacokinetic (PBPK) Model. Int. J. Pharm..

[7] Pepin X.J.H., Moir A.J., Mann J.C., Sanderson N.J., Barker R., Meehan E., Plumb A.P., Bailey G.R., Murphy D.S., Krejsa C.M. (2019). Bridging in Vitro Dissolution and in Vivo Exposure for Acalabrutinib. Part II. A Mechanistic PBPK Model for IR Formulation Comparison, Proton Pump Inhibitor Drug Interactions, and Administration with Acidic Juices. Eur. J. Pharm. Biopharm..

[8] Heikkinen A.T., Baneyx G., Caruso A., Parrott N. (2012). Application of PBPK Modeling to Predict Human Intestinal Metabolism of CYP3A Substrates—An Evaluation and Case Study Using GastroPlusTM. Eur. J. Pharm. Sci..

[9] Kambayashi A. (2023). In Silico Modeling Approaches Coupled with In Vitro Characterization in Predicting In Vivo Performance of Drug Delivery System Formulations. Mol. Pharm..

[10] Li J., Spivey N., Silchenko S., Gonzalez-Alvarez I., Bermejo M., Hidalgo I.J. (2021). A Differential Equation Based Modelling Approach to Predict Supersaturation and in Vivo Absorption from in Vitro Dissolution-Absorption System (Idas2) Data. Eur. J. Pharm. Biopharm..

[11] McFarland J.W., Avdeef A., Berger C.M., Raevsky O.A. (2001). Estimating the Water Solubilities of Crystalline Compounds from Their Chemical Structures Alone. J. Chem. Inf. Comput. Sci..

[12] Vertzoni M., Dressman J., Butler J., Hempenstall J., Reppas C. (2005). Simulation of Fasting Gastric Conditions and Its Importance for the In Vivo Dissolution of Lipophilic Compounds. Eur. J. Pharm. Biopharm..

[13] Jantratid E., Janssen N., Reppas C., Dressman J.B. (2008). Dissolution Media Simulating Conditions in the Proximal Human Gastrointestinal Tract: An Update. Pharm. Res..

[14] Johnsson G., Regärdh C., Sölvell L. (1975). Combined Pharmacokinetic and Pharmacodynamic Studies in Man of the Adrenergic β 1 -receptor Antagonist Metoprolol. Acta Pharmacol. Toxicol..

[15] Nielsen-Kudsk F., Pedersen A.K. (1979). Pharmacokinetics of Dipyridamole. Acta Pharmacol. Toxicol..

[16] Li J., Bukhtiyarov Y., Spivey N., Force C., Hidalgo C., Huang Y., Owen A.J., Hidalgo I.J. (2020). In Vitro and In Vivo Assessment of the Potential of Supersaturation to Enhance the Absorption of Poorly Soluble Basic Drugs. J. Pharm. Innov..

[17] Noyes A.A., Whitney W.R. (1897). The Rate of Solution of Solid Substances in Their Own Solutions. J. Am. Chem. Soc..

[18] Takano R., Sugano K., Higashida A., Hayashi Y., Machida M., Aso Y., Yamashita S. (2006). Oral Absorption of Poorly Water-Soluble Drugs: Computer Simulation of Fraction Absorbed in Humans from a Miniscale Dissolution Test. Pharm. Res..

[19] Jakubiak P., Wagner B., Grimm H.P., Petrig-Schaffland J., Schuler F., Alvarez-Sánchez R. (2016). Development of a Unified Dissolution and Precipitation Model and Its Use for the Prediction of Oral Drug Absorption. Mol. Pharm..

[20] Hofsäss M.A., Dressman J. (2020). Suitability of the Z-Factor for Dissolution Simulation of Solid Oral Dosage Forms: Potential Pitfalls and Refinements. J. Pharm. Sci..

[21] Yano K., Masaoka Y., Kataoka M., Sakuma S., Yamashita S. (2010). Mechanisms of Membrane Transport of Poorly Soluble Drugs: Role of Micelles in Oral Absorption Processes. J. Pharm. Sci..

[22] Kataoka M., Yano K., Hamatsu Y., Masaoka Y., Sakuma S., Yamashita S. (2013). Assessment of Absorption Potential of Poorly Water-Soluble Drugs by Using the Dissolution/Permeation System. Eur. J. Pharm. Biopharm..

[23] Katneni K., Charman S.A., Porter C.J.H. (2006). Permeability Assessment of Poorly Water-soluble Compounds under Solubilizing Conditions: The Reciprocal Permeability Approach. J. Pharm. Sci..

[24] Poelma F.G.J., Breäs R., Tukker J.J., Crommelin D.J.A. (1991). Intestinal Absorption of Drugs. The Influence of Mixed Micelles on on the Disappearance Kinetics of Drugs from the Small Intestine of the Rat. J. Pharm. Pharmacol..

[25] Kambayashi A., Yasuji T., Dressman J.B. (2016). Prediction of the Precipitation Profiles of Weak Base Drugs in the Small Intestine Using a Simplified Transfer (“Dumping”) Model Coupled with In Silico Modeling and Simulation Approach. Eur. J. Pharm. Biopharm..

[26] Grimm M., Koziolek M., Saleh M., Schneider F., Garbacz G., Kühn J.-P., Weitschies W. (2018). Gastric Emptying and Small Bowel Water Content after Administration of Grapefruit Juice Compared to Water and Isocaloric Solutions of Glucose and Fructose: A Four-Way Crossover MRI Pilot Study in Healthy Subjects. Mol. Pharm..

[27] Grimm M., Scholz E., Koziolek M., Kühn J.-P., Weitschies W. (2017). Gastric Water Emptying under Fed State Clinical Trial Conditions Is as Fast as under Fasted Conditions. Mol. Pharm..

[28] Mudie D.M., Murray K., Hoad C.L., Pritchard S.E., Garnett M.C., Amidon G.L., Gowland P.A., Spiller R.C., Amidon G.E., Marciani L. (2014). Quantification of Gastrointestinal Liquid Volumes and Distribution Following a 240 ML Dose of Water in the Fasted State. Mol. Pharm..

[29] US FDA (2014). Guidance for Industry: Bioavailability and Bioequivalence Studies Submitted in NDAs or INDs—General Considerations; DRAFT GUIDANCE. https://www.fda.gov/media/88254/download.

[30] Macheras P., Reppas C., Dressman J. (1995). Biopharmaceutics of Orally Administered Drugs.

[31] Basit A.W., Newton J.M., Short M.D., Waddington W.A., Ell P.J., Lacey L.F. (2001). The Effect of Polyethylene Glycol 400 on Gastrointestinal Transit: Implications for the Formulation of Poorly-Water Soluble Drugs. Pharm. Res..

[32] Yu L.X. (1999). An Integrated Model for Determining Causes of Poor Oral Drug Absorption. Pharm. Res..

[33] Lennernäs H. (2007). Intestinal Permeability and Its Relevance for Absorption and Elimination. Xenobiotica.

[34] Higashino H., Hasegawa T., Yamamoto M., Matsui R., Masaoka Y., Kataoka M., Sakuma S., Yamashita S. (2014). In Vitro—In Vivo Correlation of the Effect of Supersaturation on the Intestinal Absorption of BCS Class 2 Drugs. Mol. Pharm..

[35] Matsui K., Tsume Y., Amidon G.E., Amidon G.L. (2015). In Vitro Dissolution of Fluconazole and Dipyridamole in Gastrointestinal Simulator (GIS), Predicting in Vivo Dissolution and Drug–Drug Interaction Caused by Acid-Reducing Agents. Mol. Pharm..

[36] de Stoppelaar F.M., Stolk L.M.L., Beysens A.J., Stappers J.L.M., Gorgels A.P.M. (1999). The Relative Bioavailability of Metoprolol Following Oral and Rectal Administration to Volunteers and Patients. Pharm. World Sci..

[37] Galia E., Nicolaides E., Hörter D., Löbenberg R., Reppas C., Dressman J.B. (1998). Evaluation of Various Dissolution Media for Predicting In Vivo Performance of Class I and II Drugs. Pharm. Res..

[38] Incecayir T., Tsume Y., Amidon G.L. (2013). Comparison of the Permeability of Metoprolol and Labetalol in Rat, Mouse, and Caco-2 Cells: Use as a Reference Standard for BCS Classification. Mol. Pharm..

[39] Masada T., Takagi T., Minami K., Kataoka M., Takeyama S., Fujii Y., Takahashi M., Yamashita S. (2021). New Biphasic System in Side-by-Side Chambers for Testing Drug Dissolution and Permeation in Vitro (BiDP System). J. Drug Deliv. Sci. Technol..

[40] Girdhar A., Thakur P.S., Sheokand S., Bansal A.K. (2018). Permeability Behavior of Nanocrystalline Solid Dispersion of Dipyridamole Generated Using NanoCrySP Technology. Pharmaceutics.

[41] Artursson P. (1990). Epithelial Transport Of Drugs In Cell Culture. I: A Model For Studying The Passive Diffusion Of Drugs Over Intestinal Absorbtive (Caco-2) Cells. J. Pharm. Sci..

[42] Segregur D., Barker R., Mann J., Moir A., Karlsson E.M., Turner D.B., Arora S., Dressman J. (2021). Evaluating the Impact of Acid-Reducing Agents on Drug Absorption Using Biorelevant in Vitro Tools and PBPK Modeling—Case Example Dipyridamole. Eur. J. Pharm. Sci..

[43] Psachoulias D., Vertzoni M., Goumas K., Kalioras V., Beato S., Butler J., Reppas C. (2011). Precipitation in and Supersaturation of Contents of the Upper Small Intestine After Administration of Two Weak Bases to Fasted Adults. Pharm. Res..

